# Efficacy and safety of upadacitinib in patients with active ankylosing spondylitis refractory to biologic therapy: 2-year clinical and radiographic results from the open-label extension of the SELECT-AXIS 2 study

**DOI:** 10.1186/s13075-024-03412-8

**Published:** 2024-11-12

**Authors:** Xenofon Baraliakos, Désirée van der Heijde, Joachim Sieper, Robert Davies Inman, Hideto Kameda, Walter Peter Maksymowych, Ivan Lagunes-Galindo, Xianwei Bu, Peter Wung, Koji Kato, Anna Shmagel, Atul Deodhar

**Affiliations:** 1 Rheumazentrum Ruhrgebiet Herne, Ruhr-University Bochum, Claudiusstr. 45, 44649 Herne, Nordrhein- Westfalen, Germany; 2 Rheumatology, Leiden University Medical Center, Leiden, The Netherlands; 3 Gastroenterology, Infectious Diseases and Rheumatology, Charité Universitätsmedizin Berlin, Berlin, Germany; 4 Schroeder Arthritis Institute, University Health Network, University of Toronto, Toronto, ON Canada; 5 Rheumatology, Toho University, Tokyo, Japan; 6 Department of Medicine, University of Alberta, Edmonton, AB Canada; 7 Immunology, AbbVie Inc, North Chicago, IL USA; 8 Division of Arthritis & Rheumatic Diseases, Oregon Health & Science University, Portland, OR USA

**Keywords:** Ankylosing spondylitis, Radiographic axial spondyloarthritis, Biologic DMARD, Inadequate response, Open-label extension, Refractory, Upadacitinib

## Abstract

**Background:**

The efficacy and safety of upadacitinib in patients with ankylosing spondylitis (AS) and inadequate response/intolerance to biologic disease-modifying antirheumatic drugs (bDMARD-IR) were evaluated through 1 year in the SELECT-AXIS 2 study. Here, we assess 2-year efficacy, safety, and imaging outcomes in SELECT-AXIS 2.

**Methods:**

Patients who received continuous upadacitinib, and those who switched from placebo to upadacitinib at week 14, could enter the open-label extension (OLE). Efficacy endpoints included Assessment of SpondyloArthritis international Society (ASAS) and Axial Spondyloarthritis Disease Activity Score (ASDAS) responses, and changes from baseline in measures of disease activity, back pain, function, and quality of life. Radiographic progression was evaluated using the modified Stoke Ankylosing Spondylitis Spinal Score (mSASSS). As observed (AO) and AO with non-responder imputation (AO-NRI) analyses were used for binary endpoints; AO with mixed-effects model for repeated measures (AO-MMRM) for continuous endpoints; and AO-analysis of covariance for mSASSS. Treatment-emergent adverse events (TEAEs) in patients receiving ≥ 1 upadacitinib dose through week 104 are presented as events (E)/100 patient-years (PY). Subgroup analyses were performed by prior tumor necrosis factor/interleukin-17 inhibitor exposure and bDMARD lack of efficacy/intolerance.

**Results:**

Of 420 patients who entered the bDMARD-IR AS study, 409 entered the OLE, and 331 (continuous upadacitinib, *n* = 163; placebo to upadacitinib, *n* = 168) completed week 104. Improvements in efficacy measures were sustained through the OLE, with similar response rates between the continuous upadacitinib and placebo to upadacitinib groups at week 104. At week 104, 64.9% and 61.7% of patients, respectively, had achieved ASAS 40% response (AO-NRI). Mean changes from baseline were similar between the two groups at week 104 across measures (ASDAS: -2.1 and -2.0; total back pain: -4.9 and -4.6, respectively; AO-MMRM). Over 93.0% of patients showed no radiographic progression (mSASSS mean change from baseline < 2) at week 104. The overall TEAE rate was 165.2 E/100 PY, with low rates of major adverse cardiovascular and venous thromboembolic events (0.3 E/100 PY each).

**Conclusions:**

Upadacitinib efficacy, including very low rates of radiographic progression, was demonstrated through 104 weeks in treatment-refractory patients with active AS. Treatment was well tolerated, with no newly identified safety signals.

**Trial registration:**

NCT04169373.

**Supplementary Information:**

The online version contains supplementary material available at 10.1186/s13075-024-03412-8.

## Introduction

Axial spondyloarthritis (axSpA) predominantly affects the spine and sacroiliac joints (SIJs), with patients experiencing chronic back pain, spinal stiffness, and skeletal/postural changes, as well as peripheral (i.e., arthritis, enthesitis, dactylitis) and extra-musculoskeletal (i.e., uveitis, inflammatory bowel disease [IBD], psoriasis) manifestations, and reduced quality of life (QoL) [1 − 4]. AxSpA is subdivided into radiographic axSpA (r-axSpA; historically referred to as ankylosing spondylitis [AS]), characterized by definitive radiographic SIJ damage; and non-radiographic axSpA (nr-axSpA), which may be an earlier manifestation of a disease continuum, in which patients may progress to demonstrate detectable radiographic findings [1, 5]. “r-axSpA” and “AS” are considered clinically synonymous and can be used interchangeably [6]; “AS” is used throughout this article for consistency with previously published papers on this trial [7, 8].

The primary goal of axSpA treatment is to control symptoms and underlying inflammation, prevent progressive structural damage, and maximize long-term health-related QoL; however, treatment options for axSpA are more limited compared with other rheumatic diseases [5, 9, 10]. Current guidelines recommend treatment with a biologic disease-modifying antirheumatic drug (bDMARD) (tumor necrosis factor [TNF] inhibitor or interleukin [IL]-17 inhibitor) or a Janus kinase (JAK) inhibitor for patients with persistently high disease activity [5]; however, not all patients respond favorably to these drugs [11 − 19]. Switching to another bDMARD or JAK inhibitor is recommended in patients with intolerance or insufficient response to previous therapy [5, 9, 11, 20].

Upadacitinib, an oral reversible JAK inhibitor, has shown efficacy and an acceptable safety profile in patients with AS in the SELECT-AXIS 1 and 2 studies, including in patients with inadequate response (IR) to a bDMARD through 1 year in SELECT-AXIS 2 [7, 8, 21]. In this study, upadacitinib 15 mg once daily demonstrated sustained improvement up to week 52 in bDMARD-IR patients with active AS, with similar efficacy observed between patients on continuous upadacitinib and those who switched from placebo to upadacitinib after week 14. Treatment was well tolerated, consistent with the known safety profile of upadacitinib, with no new safety risks identified through week 52 [7]. Here, we present the final analysis of the open-label extension (OLE) phase of SELECT-AXIS 2, undertaken to evaluate 2-year efficacy, safety, and radiographic progression in bDMARD-IR patients with active AS treated with upadacitinib.

## Methods

### Study design

SELECT-AXIS 2 (NCT04169373) is a global, phase 3, multicenter trial that comprises two independent studies for patients with active axSpA, including bDMARD-IR AS and nr-axSpA. The bDMARD-IR AS study design has been described previously [7, 8]. Here, we present data through week 104 of the OLE of the bDMARD-IR AS study. The study is being conducted according to the International Council for Harmonization guidelines, local regulations and guidelines governing clinical study conduct, and the Declaration of Helsinki. All patients provided written informed consent, and the study protocol and consent forms were approved by an institutional review board or independent ethics committee at each study site.

### Patients

Patient characteristics have been described previously [7, 8]. Briefly, patients were aged ≥ 18 years, had a diagnosis of AS and fulfilled the modified New York criteria based on central reading of SIJ radiographs obtained at screening [22], and had active disease at baseline (defined as Bath Ankylosing Spondylitis Disease Activity Index [BASDAI] score ≥ 4 and patient’s assessment of total back pain score of ≥ 4 on a 0–10 numeric rating scale [NRS]). Eligible patients were required to have: IR to at least two non-steroidal anti-inflammatory drugs (NSAIDs) (or intolerance/contraindication to NSAIDs); IR to prior bDMARD therapy (defined as discontinuation of TNF inhibitor or IL-17 inhibitor therapy due to lack of efficacy [after ≥ 12 weeks of treatment at an adequate dose] based on the investigators’ assessment; or intolerance to bDMARDs [irrespective of treatment duration]). Patients on a stable dose of concomitant oral corticosteroids or NSAIDs for at least 14 days prior to the baseline visit, or concomitant conventional synthetic DMARDs (csDMARDs) for at least 28 days prior to the baseline visit, were considered eligible. Prior exposure to two bDMARDs was permitted for up to 30% of patients; among patients with prior exposure to two bDMARDs, lack of efficacy to one and intolerance to another bDMARD was permitted; lack of efficacy to two bDMARDs was not permitted. Patients previously exposed to any JAK inhibitor were excluded. Patients with total spinal ankylosis (defined as fusion of ≥ 5 C2–T1 or T12–S1 spinal segments) were also excluded.

### Study treatment

Patients were randomized 1:1 in a blinded manner to receive either oral upadacitinib 15 mg once daily or placebo for 14 weeks, after which all patients in the placebo group were switched to upadacitinib 15 mg once daily.

### Efficacy

Efficacy endpoints assessed through week 104 included: Assessment of SpondyloArthritis international Society (ASAS) responses, including proportion of patients achieving relative improvement of ≥ 40% in three out of the four ASAS domains without worsening in the remaining domain (ASAS40; primary efficacy endpoint at week 14), relative improvement of ≥ 20% in three out of the four ASAS domains without worsening in the remaining domain (ASAS20), and ASAS partial remission (ASAS PR; absolute score of ≤ 2 units for each of the four ASAS domains); Axial Spondyloarthritis Disease Activity Score (ASDAS) responses, including proportion of patients achieving ASDAS low disease activity (LDA; < 2.1), ASDAS inactive disease (ID; < 1.3), ASDAS major improvement (MI; ≥ 2.0-point decrease from baseline), and ASDAS clinically important improvement (CII; ≥ 1.1-point decrease from baseline); proportion of patients achieving ≥ 50% improvement in BASDAI (BASDAI50); change from baseline in patient’s assessment of total back pain and nocturnal back pain; and change from baseline in measures of function (Bath Ankylosing Spondylitis Functional Index [BASFI]), mobility (linear Bath Ankylosing Spondylitis Metrology Index [BASMI]), enthesitis (Maastricht Ankylosing Spondylitis Enthesitis Score [MASES] in patients with enthesitis [i.e., MASES > 0] at baseline), QoL (ASAS Health Index [HI] and Ankylosing Spondylitis QoL [ASQoL] score), and inflammation (high-sensitivity C-reactive protein [hsCRP]).

Additional efficacy endpoints included mean change from baseline in fatigue/tiredness (BASDAI Question 1; NRS 0–10), morning stiffness severity and duration (BASDAI Questions 5 and 6; NRS 0–10), patient’s global assessment of pain (NRS 0–10), patient’s global assessment of disease activity (NRS 0–10), Functional Assessment of Chronic Illness Therapy-Fatigue (FACIT-F), tender joint count out of 68 joints, and swollen joint count out of 66 joints.

Radiologic endpoints included: proportion of patients with no radiographic progression (defined as change from baseline in modified Stoke Ankylosing Spondylitis Spinal Score [mSASSS] < 2) at week 104; and change from baseline in mSASSS and in SpondyloArthritis Research Consortium of Canada (SPARCC) scores of the spine [23] and SIJs [24]. Conventional radiographs of the spine and pelvis were taken at baseline and at week 104. Magnetic resonance imaging (MRI) of the spine and SIJs was performed at baseline, week 14, and week 104. For the imaging analysis, baseline, week 14, and week 104 images were read together in a single reading session. Each MRI or conventional radiograph was independently assessed by two primary readers blinded to the treatment arm, time point, patient- and site-identifying information. Adjudication by a third reader was employed to resolve discrepancies between the primary readers. Adjudication was triggered by differences between the primary readers’ change scores that exceeded a defined threshold based on mean absolute differences [25, 26]. For the reading of baseline and week 14 MRIs, the adjudication trigger was ≥ 14 for MRI of the spine and ≥ 8 for MRI of the SIJs. For the reading of baseline and week 104 MRIs, the adjudication trigger was ≥ 15 for MRI of the spine and ≥ 9 for MRI of the SIJs. For change from baseline in mSASSS, the adjudication trigger was a difference of ≥ 5 mSASSS points for a given patient. Change from baseline was calculated using the average scores of two primary readers or, for adjudicated cases, the average of the two closest scores of the three readings (primary readers and adjudicator). The two closest scores were selected based on change from baseline at the week 104 visit. Cumulative probability plots were also generated to illustrate changes in SPARCC scores and mSASSS at week 104 by treatment group. The probability of progression was modeled as a function of change from baseline to week 104 in imaging scores using an ordinal logistic regression model.

### Safety

Safety was assessed through week 104 by reporting of treatment-emergent adverse events (TEAEs), defined as any adverse event (AE) with an onset date after the first dose and ≤ 30 days after the last dose of the study drug, including patients who switched from placebo to upadacitinib at week 14. TEAEs, including serious AEs (SAEs), AEs leading to discontinuation of the study drug, and AEs of special interest (AESIs), are presented as exposure-adjusted event rates (EAERs; events/100 patient-years [E/100 PY]) and exposure-adjusted incidence rates (EAIRs; n/100 PY). Blinded evaluation of all major adverse cardiovascular events (MACE) and venous thromboembolic events (VTE) was completed by an independent cardiovascular adjudication committee. Laboratory assessments, including hemoglobin, lymphocyte count, neutrophil count, and serum levels of alanine transaminase (ALT), aspartate transaminase (AST), and creatinine, were conducted through week 104 and are presented as the number and proportion of patients with potentially clinically significant values at week 104 (grade 3 or 4 based on the National Cancer Institute Common Terminology Criteria for AEs, version 4.0) [27].

### Statistical analysis

For binary efficacy endpoints, as observed (AO) and AO with non-responder imputation (AO-NRI) analyses are presented. AO-NRI uses all observed data regardless of premature discontinuation of study drug or use of rescue therapy, with patients with missing data categorized as non-responders.

Continuous efficacy endpoints are presented as change from baseline estimated from mixed-effects model for repeated measures (MMRM; based on AO data), and AO data (without imputation). Data on radiographic progression by mSASSS are presented as least-squares (LS) mean change from baseline (AO-analysis of covariance [ANCOVA]).

The safety analysis set included all patients who received ≥ 1 dose of upadacitinib 15 mg once daily at any point in the study up to their week 104 visit. Laboratory data were assessed in patients who received ≥ 1 dose of upadacitinib 15 mg once daily at any point in the study up to their week 104 visit and had both baseline and post-baseline values available.

Post hoc analyses for efficacy endpoints at week 104 were performed in subgroups of patients with prior exposure to a TNF inhibitor or an IL-17 inhibitor, and in those who discontinued prior bDMARD therapy due to lack of efficacy or intolerance.

## Results

### Patient disposition and baseline characteristics

**Fig. 1:**
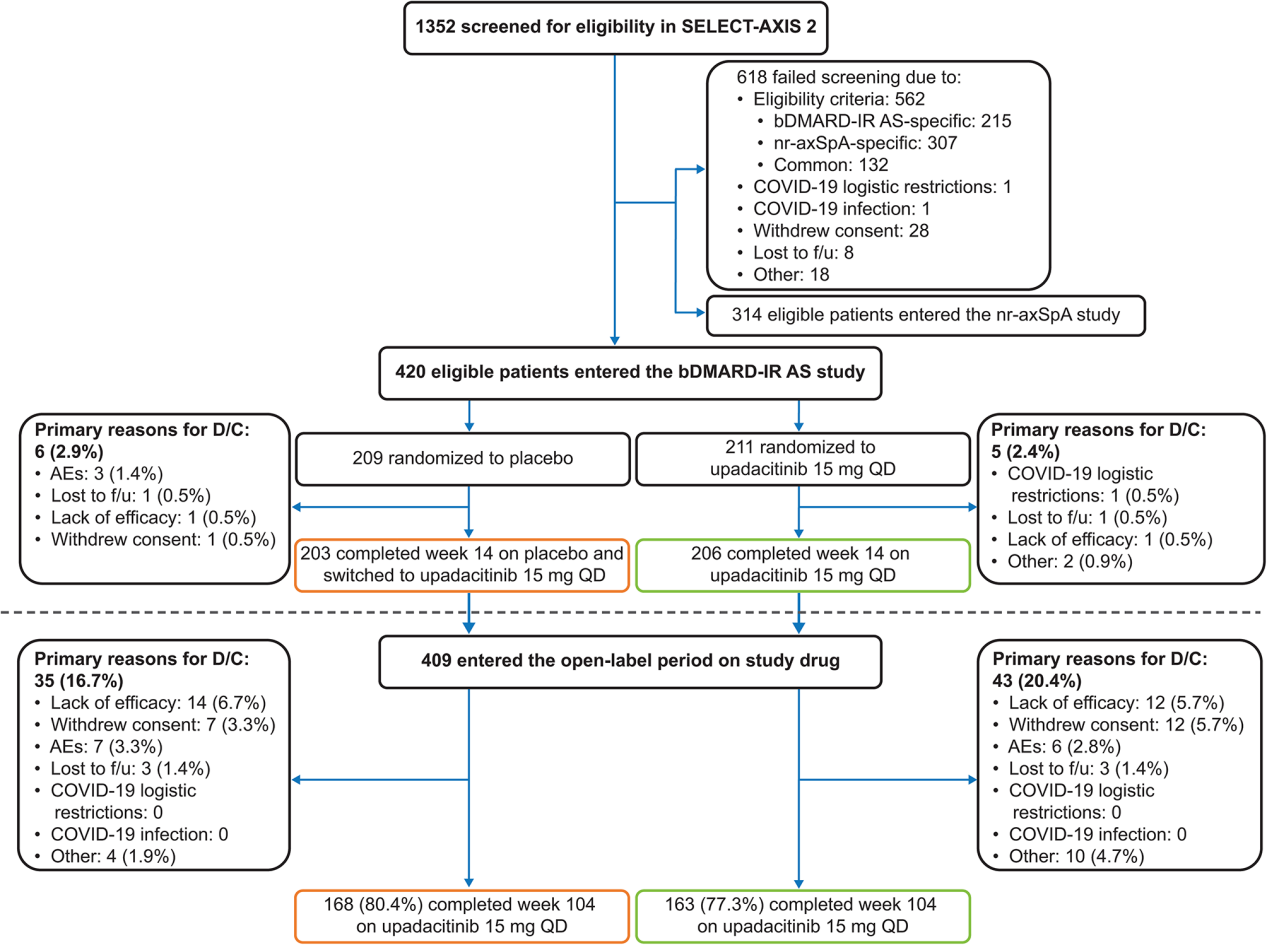
Patient disposition through week 104 *AE* adverse event, *AS* ankylosing spondylitis, *bDMARD* biologic disease-modifying antirheumatic drug, *D/C* discontinuation, *f/u* follow-up, *IR* inadequate response, *nr-axSpA* non-radiographic axial spondyloarthritis, *QD* once daily

Of 420 patients who entered the bDMARD-IR AS study (*n* = 211 initially randomized to upadacitinib 15 mg once daily and *n* = 209 initially randomized to placebo), 409 patients entered the OLE period on study drug (206/211 who completed week 14 on upadacitinib 15 mg once daily [continuous upadacitinib group], and 203/209 who completed week 14 on placebo and switched to upadacitinib 15 mg once daily [placebo to upadacitinib group]). In total, 331 (78.8%) patients completed 104 weeks of treatment (*n* = 163 and *n* = 168 in the continuous upadacitinib and placebo to upadacitinib groups, respectively) (Fig. 1). A total of 78 patients discontinued the study drug in the OLE period (*n* = 43 [20.4%] in the continuous upadacitinib and *n* = 35 [16.7%] in the placebo to upadacitinib groups). The most common primary reason for discontinuation in both groups was lack of efficacy (continuous upadacitinib: *n* = 12; placebo to upadacitinib: *n* = 14), followed by withdrawal of consent (continuous upadacitinib: *n* = 12; placebo to upadacitinib: *n* = 7), and AEs (continuous upadacitinib: *n* = 6; placebo to upadacitinib: *n* = 7) (Fig. 1).

Patient demographics and baseline disease characteristics have been reported previously, and were generally well balanced between the two study arms [7, 8]. The majority of patients had received prior TNF inhibitor therapy (continuous upadacitinib group: *n* = 181 [85.8%]; placebo to upadacitinib group: *n* = 183 [87.6%]) compared with IL-17 inhibitor therapy (continuous upadacitinib group: *n* = 38 [18.0%]; placebo to upadacitinib group: *n* = 36 [17.2%]). The most commonly reported cause of prior bDMARD failure in both groups was lack of efficacy (continuous upadacitinib group: *n* = 166 [78.7%]; placebo to upadacitinib group: *n* = 159 [76.1%]).

Similar proportions of patients in the two groups were receiving concomitant medications at week 104: in total, 81.7% of patients were receiving concomitant NSAIDs, 33.6% were receiving concomitant csDMARDs, 15.7% were receiving concomitant oral corticosteroids, and 12.9% were receiving concomitant opioids.

### Efficacy

**Fig. 2:**
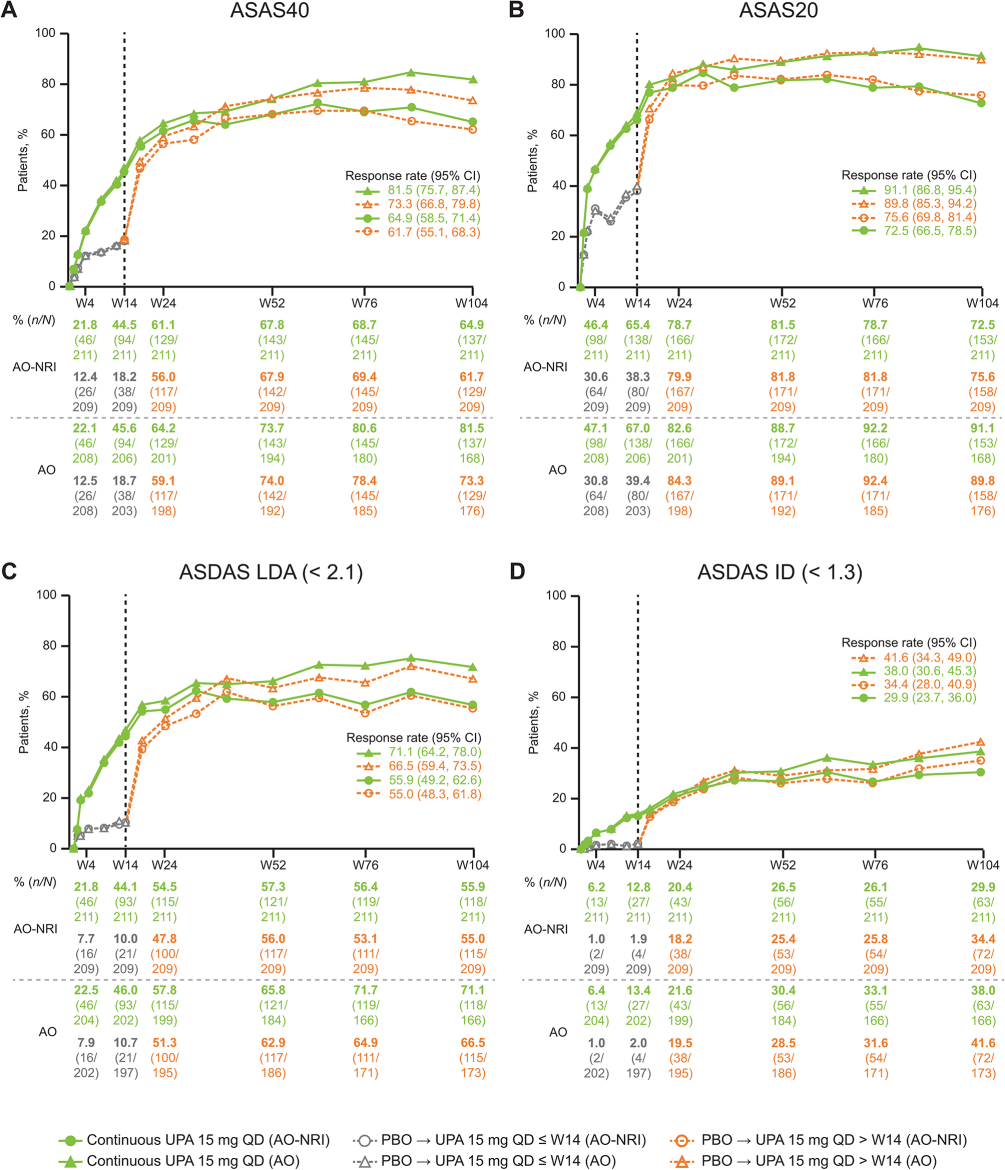
Proportions of patients achieving ASAS40 (**A**), ASAS20 (**B**), ASDAS LDA (**C**), and ASDAS ID (**D**) responses through week 104 (AO-NRI and AO) Patients who were initially randomized to placebo were switched to open-label upadacitinib at week 14 *AO* as observed, *ASAS* Assessment of SpondyloArthritis international Society, *ASAS40* ≥ 40% improvement in three out of the four ASAS domains without worsening in the remaining domain, *ASAS20* ≥ 20% improvement in three out of the four ASAS domains without worsening in the remaining domain, *ASDAS* Axial Spondyloarthritis Disease Activity Score, *CI* confidence interval, *ID* inactive disease, *LDA* low disease activity, *NRI* non-responder imputation, *PBO* placebo, *QD* once daily, *UPA* upadacitinib, *W* week

The proportion of patients in the continuous upadacitinib group achieving ASAS40 response increased further from 44.5% at week 14 to 64.9% at week 104 (AO-NRI; Fig. 2). The proportion of patients in the placebo to upadacitinib group achieving ASAS40 increased steeply from 18.2% at week 14 to 56.0% at week 24 (AO-NRI) following switch to upadacitinib, and continued to increase through week 104. At week 104, a similar proportion of patients in the placebo to upadacitinib group had achieved ASAS40 (61.7%) as in the continuous upadacitinib group (AO-NRI; Fig. 2). ASAS20 results followed a similar pattern, with the proportion of patients achieving ASAS20 in the continuous upadacitinib group increasing from 65.4% at week 14 to 72.5% at week 104, and the proportion of patients in the placebo to upadacitinib group increasing from 38.3% at week 14 to 75.6% at week 104 (AO-NRI; Fig. 2).

A similar pattern was observed for additional efficacy measures, including the proportion of patients achieving ASDAS LDA, ASDAS ID (Fig. 2), ASAS PR, BASDAI50 (Supplementary Fig. S1), ASDAS MI, and ASDAS CII (Supplementary Table S1), with a comparable magnitude of response between the continuous upadacitinib and placebo to upadacitinib groups at week 104.

**Fig. 3:**
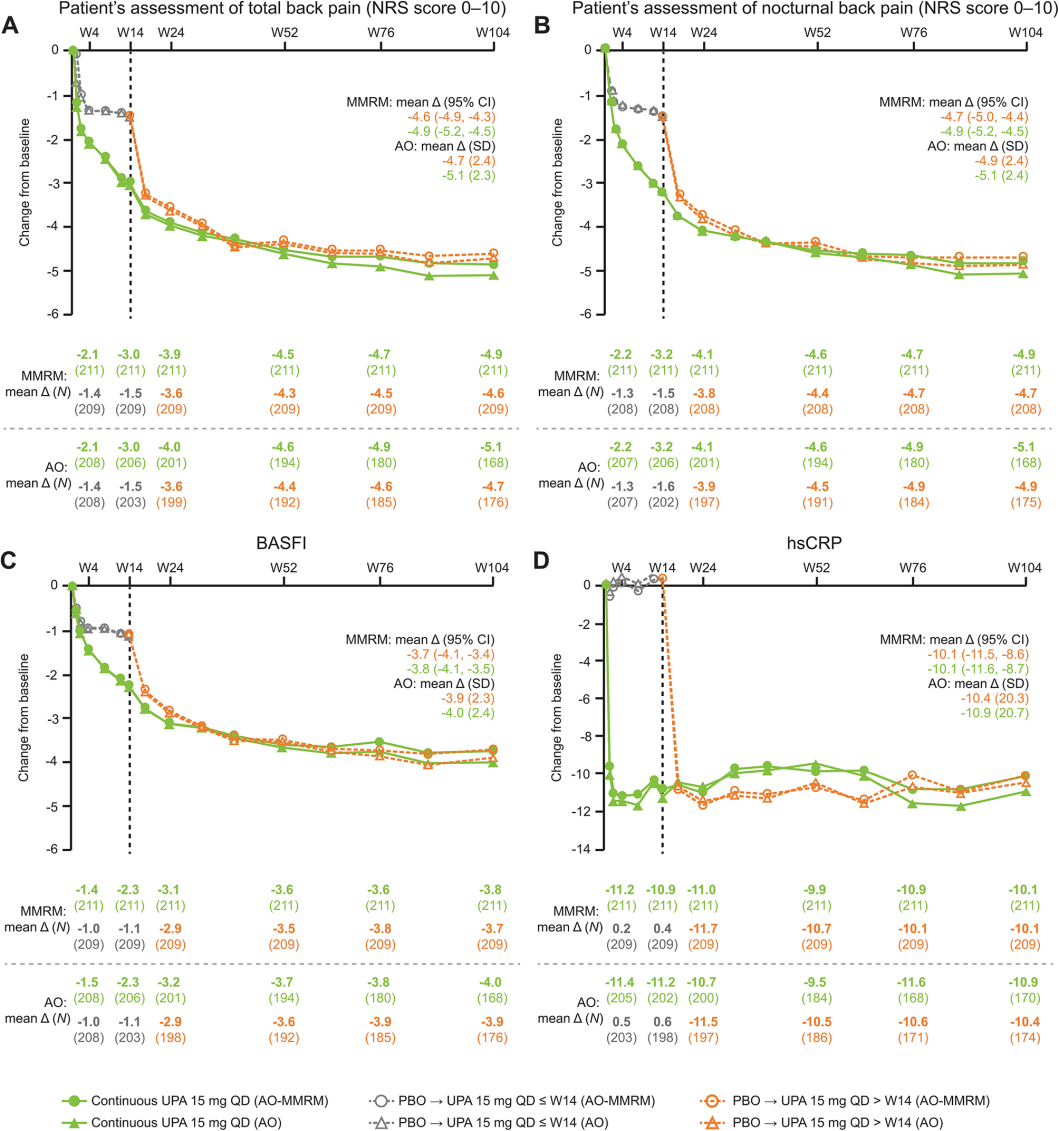
Mean change from baseline in total back pain (**A**), nocturnal back pain (**B**), BASFI (**C**), and hsCRP (**D**) through week 104 (AO-MMRM and AO) Patients who were initially randomized to placebo were switched to open-label upadacitinib at week 14. *Δ* change, *AO* as observed, *BASFI* Bath Ankylosing Spondylitis Functional Index, *CI* confidence interval, *hsCRP* high-sensitivity C-reactive protein, *MMRM* mixed-effects model for repeated measures, *NRS* numeric rating scale, *PBO* placebo, *QD* once daily, *SD* standard deviation, *UPA* upadacitinib, *W* week

Improvements (change from baseline) from week 14 to week 104 were also observed in pain assessment measures, including patient’s assessment of total back pain and nocturnal back pain (Fig. 3), and patient’s global assessment of pain (Supplementary Table S1). Similarly, improvements from week 14 through week 104 were observed in measures of function (BASFI), mobility (BASMI), enthesitis (MASES), QoL (ASQoL and ASAS HI), and inflammation (hsCRP) in both groups (Fig. 3 and Supplementary Fig. S2). Additional efficacy results at week 104, including improvements in components of the BASDAI questionnaire, patient’s global assessment of disease activity, FACIT-F, and affected joint counts, are summarized in Supplementary Table S1.

**Fig. 4:**
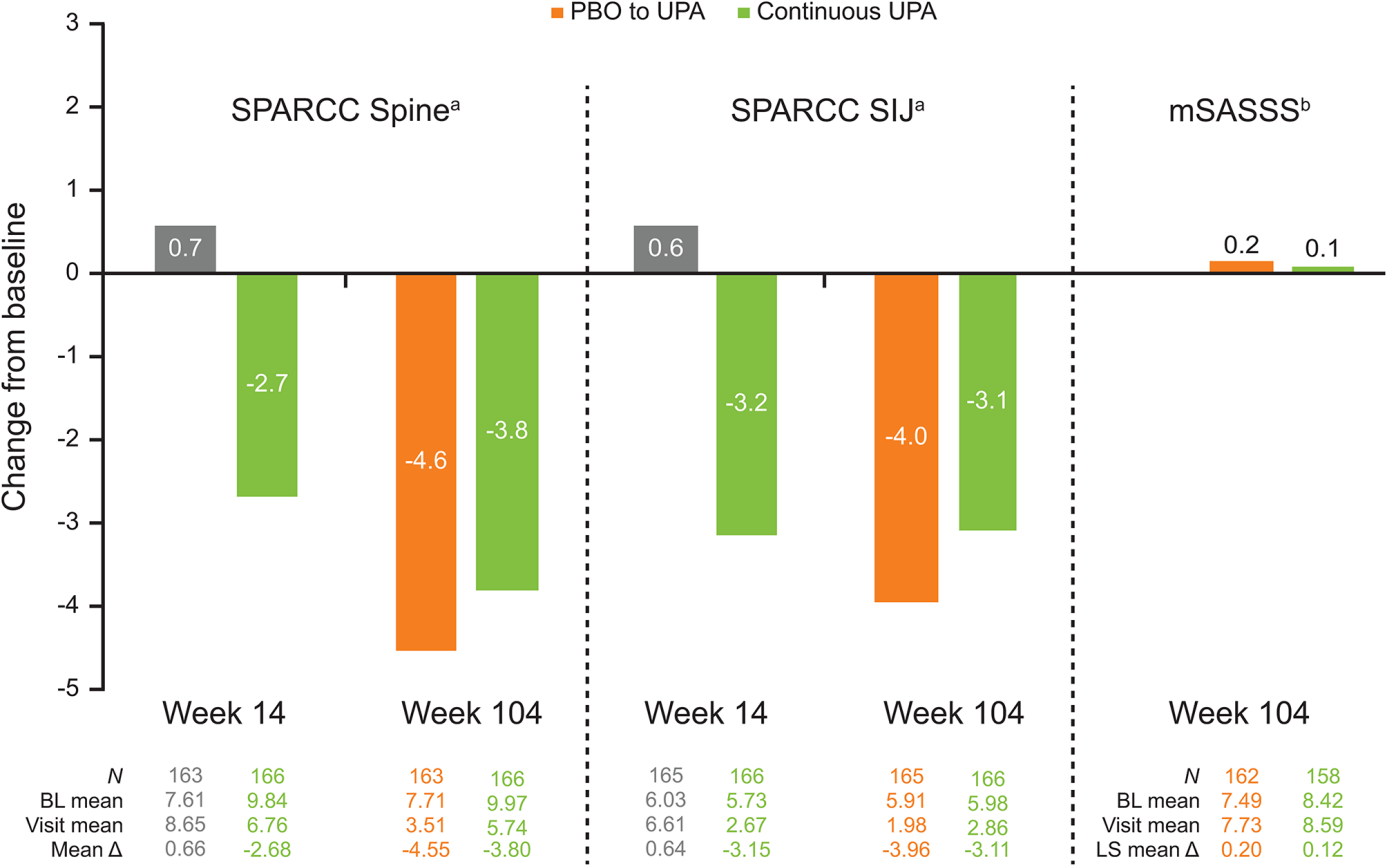
Mean change from baseline in imaging scores through week 104 (AO-MMRM and AO-ANCOVA) ^a^AO-MMRM. ^b^AO-ANCOVA. SPARCC scores were based on magnetic resonance imaging from baseline, week 14, and week 104 (2-year reading), and from premature discontinuation visits or unscheduled visits that occurred after week 76 and prior to week 104. mSASSS was assessed in patients with available X-rays of the spine from baseline and week 104, and from premature discontinuation visits that occurred after week 76 and prior to week 104 *Δ* change, *ANCOVA* analysis of covariance, *AO* as observed, *BL* baseline, *LS* least-squares, *MMRM* mixed-effects model for repeated measures, *mSASSS* modified Stoke Ankylosing Spondylitis Spinal Score, *PBO* placebo, *SIJ* sacroiliac joint, *SPARCC* SpondyloArthritis Research Consortium of Canada, *UPA* upadacitinib

The majority of patients showed no radiographic progression through week 104, with 94.9% of patients in the continuous upadacitinib group and 93.8% of patients in the placebo to upadacitinib group showing a change from baseline in mSASSS of < 2 at week 104 (Supplementary Table S1). The LS mean change from baseline (baseline mean) in mSASSS at week 104 was 0.1 (8.4) in the continuous upadacitinib group and 0.2 (7.5) in the placebo to upadacitinib group (AO-ANCOVA) (Fig. 4). In the continuous upadacitinib group, the mean change from baseline (baseline mean) in the MRI SPARCC score for inflammation in the spine further decreased from -2.7 (9.8) at week 14 to -3.8 (10.0) at week 104, while the mean change from baseline in the MRI SPARCC score for inflammation in the SIJs did not differ markedly between week 14 (-3.2 [5.7]) and week 104 (-3.1 [6.0]) (AO-MMRM). Patients in the placebo to upadacitinib group showed a similar magnitude of response for both the spinal and SIJ SPARCC scores (change from baseline of -4.6 and -4.0, respectively) at week 104 (Fig. 4). The cumulative probability plots of change in imaging scores showed that both the spinal and SIJ SPARCC scores improved or remained stable in most patients, with minor differences between the two groups; mSASSS remained stable in most patients, with no notable differences between the two treatment groups (Supplementary Fig. S3).

On subgroup analysis by prior exposure to a TNF inhibitor or IL-17 inhibitor and prior lack of efficacy or intolerance to bDMARDs, responses were generally similar across endpoints compared with the overall population (Supplementary Tables S2 and S3).

### Safety

**Fig. 5:**
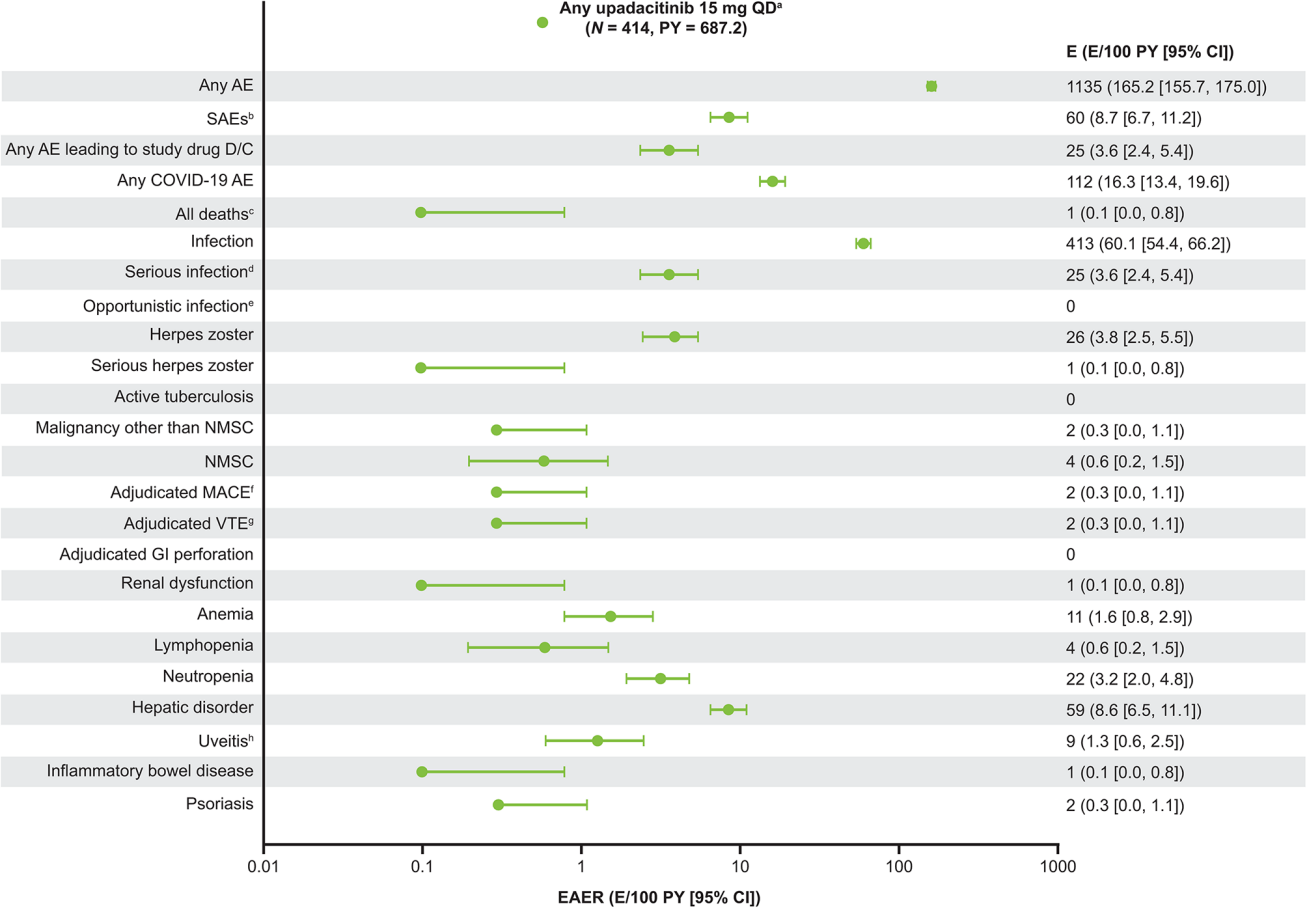
TEAEs through week 104 ^a^All patients who received ≥ 1 dose of upadacitinib 15 mg once daily. ^b^The most commonly reported SAEs were COVID-19 pneumonia (10 E; 1.5 E/100 PY) and COVID-19 (4 E; 0.6 E/100 PY). ^c^One patient died due to polytrauma before week 52. ^d^Fourteen of the 25 reported serious infections were COVID-19 events. ^e^Excluding tuberculosis and herpes zoster. ^f^Defined as cardiovascular death (includes acute myocardial infarction, sudden cardiac death, heart failure, cardiovascular procedure-related death, death due to cardiovascular hemorrhage, fatal stroke, pulmonary embolism, and other cardiovascular causes), non-fatal myocardial infarction, and non-fatal stroke. ^g^Includes deep vein thrombosis and pulmonary embolism (fatal and non-fatal). ^h^Includes uveitis, iritis, and iridocyclitis *AE* adverse event, *CI* confidence interval, *D/C* discontinuation, *E* event, *EAER* exposure-adjusted event rate, *GI* gastrointestinal, *MACE* major adverse cardiovascular events, *NMSC* non-melanoma skin cancer, *PY* patient-years, *QD* once daily, *SAE* serious AE, TEAE treatment-emergent AE, *VTE* venous thromboembolic events

Safety through week 104 was assessed in a total of 414 patients (687.2 PY) who received ≥ 1 dose of upadacitinib, with a mean exposure to upadacitinib of 606.3 days. The overall EAER of any AE was 165.2 E/100 PY, the EAER of SAEs was 8.7 E/100 PY, and the EAER of AEs leading to discontinuation of the study drug was 3.6 E/100 PY (Fig. 5). One reported death (0.1 E/100 PY) occurred before week 52 and was due to polytrauma [7]. EAIRs for TEAEs through week 104 are summarized in Supplementary Figure S4.

The EAERs of serious infection and herpes zoster infection were 3.6 and 3.8 E/100 PY, respectively (Fig. 5). There was one serious case of herpes zoster (0.1 E/100 PY). There were no reported cases of opportunistic infection or active tuberculosis. The most commonly reported SAEs were COVID-19 pneumonia (1.5 E/100 PY) and COVID-19 (0.6 E/100 PY). A total of five SAEs in three patients resulted in discontinuation of upadacitinib; six SAEs that occurred in six patients were considered as having a reasonable possibility of being related to upadacitinib; and nine SAEs in six patients were considered by the investigator to be life threatening, only one of which (one case of pulmonary embolism) was considered to have a reasonable possibility of being related to upadacitinib (Supplementary Table S4).

The EAERs of malignancy (excluding non-melanoma skin cancer), MACE, and VTE through week 104 were 0.3 E/100 PY each (Fig. 5). Details on these events are provided in Supplementary Table S5.

There were nine events of uveitis (1.3 E/100 PY), three of which were recurrent in patients with a history of uveitis; five of the nine events (two cases of iridocyclitis and three cases of uveitis) were serious, and treatment was interrupted in two cases of uveitis during the OLE. One event (0.1 E/100 PY) of new-onset IBD (Crohn’s disease) of moderate severity was reported. None of these events led to permanent discontinuation of upadacitinib.

The most common potentially clinically significant hematologic laboratory abnormalities observed with upadacitinib treatment were grade 3 reductions in neutrophil count (11/413 [2.7%]) and grade 3 reductions in lymphocyte count (5/413 [1.2%]), all of which resolved without additional treatment. A total of four events of grade 3 neutropenia in three patients had a reasonable possibility of being associated with the study drug (treatment was interrupted in one patient and permanently withdrawn in another patient who experienced two events). Most of the neutropenia events were transient and returned to baseline/grade 0 values within 1 week to 5 months without permanent discontinuation of the study drug. Decreases in neutrophil count were not found to be associated with increased risk of serious infection. One event of grade 3 lymphopenia was considered to have a reasonable possibility of being associated with the study drug (treatment interrupted). Grade 3 increases were also observed in serum levels of ALT and AST (6/413 [1.5%] each). There were no grade 4 abnormalities in any of the assessed laboratory parameters (Supplementary Table S6). Laboratory abnormalities leading to treatment discontinuation included one event of anemia in one patient (0.1 E/100 PY; not associated with the study drug), and one event each of ALT elevation (0.1 E/100 PY) and AST elevation (0.1 E/100 PY) in the same patient (reasonable possibility of being associated with the study drug).

Overall, the safety results were consistent with the known safety profile of upadacitinib [28], with no new significant safety risks identified through 104 weeks.

## Discussion

We have presented 2-year data on the safety and efficacy of upadacitinib 15 mg once daily, including its effect on radiographic progression, in bDMARD-IR patients with active AS in the SELECT-AXIS 2 study. Of patients who entered the OLE, > 80% completed week 104 of treatment. The efficacy of upadacitinib demonstrated in the initial double-blind period and through week 52 [7] was sustained with longer-term treatment through week 104 of the OLE across a wide range of clinically relevant domains, including disease activity, pain, function, enthesitis, mobility, and QoL, with a similar magnitude of response between the continuous upadacitinib and placebo to upadacitinib groups at week 104.

The discontinuation rate of upadacitinib observed in this study is lower than that previously reported for other JAK inhibitors, and its sustained efficacy profile is in line with other long-term studies of bDMARDs and JAK inhibitors in rheumatologic indications [29–33].

Improvements in efficacy outcomes at week 104 were also observed in the subgroups of patients analyzed by prior exposure to a TNF inhibitor or IL-17 inhibitor and prior lack of efficacy or intolerance to bDMARD treatment. Overall, responses were similar in the subgroups compared with the overall population; however, further interpretations or conclusions are limited due to the particularly small patient numbers in the subgroup with prior IR to an IL-17 inhibitor.

In the continuous upadacitinib group, active MRI inflammation of the spine further improved at week 104 compared with week 14, while the level of reduction of active MRI inflammation of the SIJs observed at week 14 was maintained through week 104. In addition, > 93% of patients in both groups showed no radiographic progression (defined as change from baseline in mSASSS < 2) at week 104, with an LS mean change from baseline in mSASSS of ≤ 0.2 for both groups. These observations may be due to the inhibitory effects of upadacitinib on adaptive and innate immune pathways, and the downregulation of the protein expression of pro-inflammatory biomarkers (e.g., IL-6, matrix metalloproteinase [MMP]-1, MMP-3, and IL-17 receptor A) that are markedly elevated in bDMARD-IR AS [34]. These findings appear more promising than those previously reported for bDMARDs [30, 35–37]. No radiographic progression was observed in 79% of patients treated with secukinumab, with a mean change from baseline in mSASSS of 0.5–0.7 through 104 weeks [30]; 69.7% of patients treated with infliximab showed no radiographic progression at 2 years, with a mean change from baseline in mSASSS of 0.9 [35]. The SURPASS study (lack of radiographic progression defined as change from baseline in mSASSS ≤ 0.5), which compared spinal radiographic progression with secukinumab versus an adalimumab biosimilar, reported an LS mean change from baseline in mSASSS of 0.54–0.72 [37]. The results of the present study are particularly relevant for the management of patients with AS refractory to previous treatment, as radiographic progression is associated with increased disability, reduced QoL, and higher healthcare costs [38]. However, comparing the results of different studies must be approached with caution due to possible differences in methodology, patient demographics, and disease characteristics at baseline. In particular, higher mSASSS at baseline is considered as an important risk factor for radiographic progression [39]; in the aforementioned studies, the baseline mSASSS ranged between 8.0 and 17.7, while in the present study the baseline mSASSS was at the lower end of this range (7.5 in the placebo to upadacitinib group and 8.4 in the continuous upadacitinib group). Therefore, only studies performing direct comparisons between treatments, such as the SURPASS study [37], can enable reliable conclusions on the superiority of one drug over another in delaying/arresting radiographic progression.

The long-term safety results from the bDMARD-IR AS study through week 104 showed that upadacitinib 15 mg once daily was generally well tolerated, as assessed by the frequency of TEAEs, including SAEs, AEs leading to discontinuation of upadacitinib, AESIs, and laboratory assessments. The rates of malignancy, MACE, and VTE were low, and the overall results are consistent with those observed in the rheumatoid arthritis, psoriatic arthritis, nr-axSpA, and bDMARD-naïve AS clinical trial programs for upadacitinib [21, 25, 28, 40, 41], with no new safety risks identified through 104 weeks. However, caution is advised when using JAK inhibitors, including upadacitinib, for the treatment of rheumatologic diseases in patients with pre-existing cardiovascular risk factors [42 − 44].

Limitations of this study have been discussed previously [7, 8] and include: lack of a comparator arm and the open-label study design during the extension period; potential patient selection bias due to the lack of an established definition for IR; and potential survivor bias due to missing data from patients who discontinued the study prematurely.

## Conclusion

The efficacy of upadacitinib 15 mg once daily, including very low rates of radiographic progression, was demonstrated through 104 weeks in a treatment-refractory population of patients with active AS. Observed improvements were comparable between patients receiving continuous upadacitinib and those initially randomized to placebo who switched to upadacitinib after the first 14 weeks of the study. Upadacitinib 15 mg once daily was well tolerated in this patient population, with no new safety signals identified through 104 weeks. These results suggest that upadacitinib is effective as long-term therapy for treatment-refractory patients with active AS.

## Electronic supplementary material

Below is the link to the electronic supplementary material.

Supplementary Material 1

## Data Availability

AbbVie is committed to responsible data sharing regarding the clinical trials we sponsor. This includes access to anonymized, individual, and trial-level data (analysis data sets), as well as other information (e.g., protocols, clinical study reports, or analysis plans), as long as the trials are not part of an ongoing or planned regulatory submission. This includes requests for clinical trial data for unlicensed products and indications. These clinical trial data can be requested by any qualified researchers who engage in rigorous, independent scientific research, and will be provided following review and approval of a research proposal, Statistical Analysis Plan, and execution of a Data Sharing Agreement. Data requests can be submitted at any time after approval in the US and Europe and after acceptance of this manuscript for publication. The data will be accessible for 12 months, with possible extensions considered. For more information on the process or to submit a request, please visit the following link: https://www.abbvieclinicaltrials.com/hcp/data-sharing.

## Abbreviations

AE: Adverse event
AESI: AE of special interest
ALT: Alanine transaminase
ANCOVA: Analysis of covariance
AO: As observed
AS: Ankylosing spondylitis
ASAS: Assessment of SpondyloArthritis international Society
ASAS20: ≥ 20% Improvement in three out of the four ASAS domains without worsening in the remaining domain
ASAS40: ≥ 40% Improvement in three out of the four ASAS domains without worsening in the remaining domain
ASDAS: Axial Spondyloarthritis Disease Activity Score
ASQoL: Ankylosing Spondylitis QoL
AST: Aspartate transaminase
axSpA: Axial spondyloarthritis
BASDAI: Bath Ankylosing Spondylitis Disease Activity Index
BASDAI50: ≥ 50% Improvement in BASDAI
BASFI: Bath Ankylosing Spondylitis Functional Index
BASMI: Bath Ankylosing Spondylitis Metrology Index
BCC: Basal cell carcinoma
bDMARD: Biologic DMARD
BL: Baseline
CI: Confidence interval
CII: Clinically important improvement
csDMARD: Conventional synthetic DMARD
D/C: Discontinuation
DMARD: Disease-modifying antirheumatic drug
DVT: Deep vein thrombosis
E: Event
EAER: Exposure-adjusted event rate
EAIR: Exposure-adjusted incidence rate
FACIT-F: Functional Assessment of Chronic Illness Therapy-Fatigue
f/u: Follow-up
GI: Gastrointestinal
HI: Health Index
hsCRP: High-Sensitivity C-reactive protein
IBD: Inflammatory bowel disease
ID: Inactive disease
IL: Interleukin
IL-17i: IL-17 inhibitor
IR: Inadequate response
JAK: Janus kinase
LDA: Low disease activity
LS: Least-squares
MACE: Major adverse cardiovascular event
MASES: Maastricht Ankylosing Spondylitis Enthesitis Score
MI (ASDAS): Major improvement
MI: Myocardial infarction
MMP: Matrix metalloproteinase
MMRM: Mixed-effects model for repeated measures
MRI: Magnetic resonance imaging
mSASSS: Modified Stoke Ankylosing Spondylitis Spinal Score
N/A: Not applicable
NMSC: Non-melanoma skin cancer
nr-axSpA: Non-radiographic axSpA
NRI: Non-responder imputation
NRS: Numeric rating scale
NSAID: Non-steroidal anti-inflammatory drug
OLE: Open-label extension
PBO: Placebo
PR: Partial remission
PY: Patient-years
QD: Once daily
QoL: Quality of life
r-axSpA: Radiographic axSpA
SAE: Serious AE
SCC: Squamous cell carcinoma
SD: Standard deviation
SIJ: Sacroiliac joint
SPARCC: SpondyloArthritis Research Consortium of Canada
TEAE: Treatment-emergent AE
TNF: Tumor necrosis factor
TNFi: TNF inhibitor
ULN: Upper limit of normal
UPA: Upadacitinib
VTE: Venous thromboembolic event
W: Week
