# Supplementary material for: Efficacy and safety of upadacitinib in patients with active ankylosing spondylitis refractory to biologic therapy: 2-year clinical and radiographic results from the open-label extension of the SELECT-AXIS 2 study
Source: Arthritis Res Ther. 2024 Nov 12;26:197. doi: 10.1186/s13075-024-03412-8 (PMC11556075; doi:10.1186/s13075-024-03412-8)
Supplement: Supplementary file 1 — Supplementary Material 1 [file 13075_2024_3412_MOESM1_ESM.docx]

**Efficacy and safety of upadacitinib in patients with active ankylosing spondylitis refractory to biologic therapy: 2-year clinical and radiographic results from the open-label extension of the SELECT-AXIS 2 study**

Xenofon Baraliakos^1*^, Désirée van der Heijde^2^, Joachim Sieper^3^, Robert Davies Inman^4^, Hideto Kameda^5^, Walter Peter Maksymowych^6^, Ivan Lagunes-Galindo^7^, Xianwei Bu^7^, Peter Wung^7^, Koji Kato^7^, Anna Shmagel^7^ and Atul Deodhar^8^

^1^Rheumazentrum Ruhrgebiet Herne, Ruhr-University Bochum, Herne, Germany. ^2^Rheumatology, Leiden University Medical Center, Leiden, The Netherlands. ^3^Gastroenterology, Infectious Diseases and Rheumatology, Charité Universitätsmedizin Berlin, Berlin, Germany. ^4^Schroeder Arthritis Institute, University Health Network, and University of Toronto, Toronto, ON, Canada. ^5^Rheumatology, Toho University, Tokyo, Japan. ^6^Department of Medicine, University of Alberta, Edmonton, Alberta, Canada. ^7^Immunology, AbbVie Inc., North Chicago, IL, USA. ^8^Division of Arthritis & Rheumatic Diseases, Oregon Health & Science University, Portland, OR, USA.

*Correspondence:
Xenofon Baraliakos
xenofon.baraliakos@elisabethgruppe.de
Rheumazentrum Ruhrgebiet Herne, Ruhr-University Bochum, Claudiusstr. 45, 44649 Herne, Nordrhein-Westfalen, Germany
ORCID ID: 0000-0002-9475-9362

**TARGET JOURNAL**: Arthritis Research & Therapy

**ARTICLE TYPE**: Original research **Trial registration** NCT04169373

**Supplementary Information**

**Table S1** Additional efficacy results at week 104

|  | Placebo to upadacitinib  15 mg QD | | Continuous upadacitinib  15 mg QD | |
| --- | --- | --- | --- | --- |
| Patients, % | **AO-NRI  *N =* 209** | **AO**  ***N =* 173** | **AO-NRI  *N =* 211** | **AO**  ***N =* 166** |
| ASDAS major improvement (≥ 2.0-point decrease from baseline) | 44.5 | 53.8 | 48.8 | 62.0 |
| ASDAS clinically important improvement (≥ 1.1-point decrease from baseline) | 66.5 | 80.3 | 67.3 | 85.5 |
| No radiographic progression (change from baseline in mSASSS < 2) | NA | 93.8^b^ | NA | 94.9^c^ |
| Mean change from baseline | **MMRM**  ***N =* 209** | **AO (SD)**  ***N =* 176** | **MMRM**  ***N =* 211** | **AO (SD)**  ***N =* 168** |
| ASDAS | -2.04 | -2.13 (1.18)^d^ | -2.08 | -2.23 (1.04)^e^ |
| Morning stiffness severity (BASDAI Q5)^a^ | -4.78 | -4.99 (2.35) | -4.75 | -4.98 (2.58) |
| Morning stiffness duration (BASDAI Q6)^a^ | -4.15 | -4.15 (2.97) | -4.17 | -4.45 (2.90) |
| Mean of BASDAI Q5 and Q6 | -4.44 | -4.57 (2.48) | -4.45 | -4.72 (2.55) |
| Patient’s global assessment of pain^a^ | -4.66 | -4.83 (2.38) | -4.98 | -5.23 (2.25) |
| Patient’s global assessment of disease activity^a^ | -4.58 | -4.74 (2.46) | -4.89 | -5.14 (2.35) |
| Fatigue/tiredness (BASDAI Q1)^a^ | -4.02 | -4.13 (2.58) | -4.24 | -4.58 (2.45) |
| FACIT-F | 11.53^f^ | 11.87 (10.33)^d^ | 11.67^g^ | 12.07 (10.23) |
| SJC66 | -1.5^f^ | -1.5 (3.66) | -1.4^h^ | -1.2 (2.89)^d^ |
| TJC68 | -3.3^f^ | -3.3 (6.64) | -3.3^h^ | -3.4 (6.12)^d^ |

^a^NRS score 0–10. ^b^*N =* 162. ^c^*N =* 158. ^d^*N =* 173. ^e^*N =* 166. ^f^*N =* 205. ^g^*N =* 209. ^h^*N =* 206.

*AO* as observed, *ASDAS* Axial Spondyloarthritis Disease Activity Score, *BASDAI* Bath Ankylosing Spondylitis Disease Activity Index, *FACIT-F* Functional Assessment of Chronic Illness Therapy-Fatigue, *MMRM* mixed-effects model for repeated measures, *mSASSS* modified Stoke Ankylosing Spondylitis Spinal Score, *NA* not available, *NRI* non-responder imputation, *NRS* numeric rating scale, *Q* question, *QD* once daily, *SD* standard deviation, *SJC66* swollen joint count out of 66 joints (among patients with baseline SJC > 0); *TJC68* tender joint count out of 68 joints (among patients with baseline TJC > 0)

**Table S2** Subgroup analysis of selected efficacy endpoints at week 104 by type of prior bDMARD

| Subgroup | TNFi-IR | | | | IL-17i-IR | | | |
| --- | --- | --- | --- | --- | --- | --- | --- | --- |
| Treatment arm | **Placebo to upadacitinib  15 mg QD** | | **Continuous upadacitinib  15 mg QD** | | **Placebo to upadacitinib  15 mg QD** | | **Continuous upadacitinib  15 mg QD** | |
| Binary endpoints,  n (%) | **AO-NRI**  ***N* = 183** | **AO**  ***N* = 152** | **AO-NRI**  ***N* = 181** | **AO**  ***N* = 142** | **AO-NRI**  ***N* = 36** | **AO**  ***N* = 33** | **AO-NRI**  ***N* = 38** | **AO**  ***N* = 29** |
| ASAS40 | 114 (62.3) | 114 (75.0) | 115 (63.5) | 115 (81.0) | 21 (58.3) | 21 (63.6) | 24 (63.2) | 24 (82.8) |
| ASAS20 | 141 (77.0) | 141 (92.8) | 129 (71.3) | 129 (90.8) | 26 (72.2) | 26 (78.8) | 27 (71.1) | 27 (93.1) |
| ASDAS LDA (< 2.1) | 101 (55.2) | 101 (67.3)^a^ | 99 (54.7) | 99 (70.7)^b^ | 19 (52.8) | 19 (59.4)^c^ | 20 (52.6) | 20 (69.0) |
| ASDAS ID (< 1.3) | 64 (35.0) | 64 (42.7)^a^ | 54 (29.8) | 54 (38.6)^b^ | 8 (22.2) | 8 (25.0)^c^ | 10 (26.3) | 10 (34.5) |
| ASAS PR | 71 (38.8) | 71 (46.7) | 63 (34.8) | 63 (44.4) | 9 (25.0) | 9 (27.3) | 11 (28.9) | 11 (37.9) |
| BASDAI50 | 106 (57.9) | 106 (69.7) | 112 (61.9) | 112 (78.9) | 17 (47.2) | 17 (51.5) | 22 (57.9) | 22 (75.9) |

| Continuous endpoints, mean change from baseline | MMRM | AO (SD) | MMRM | AO (SD) | MMRM | AO (SD) | MMRM | AO (SD) |
| --- | --- | --- | --- | --- | --- | --- | --- | --- |
| Patient's assessment of total back pain^d^ | *N* = 183  -4.7 | *N* = 152  -4.8 (2.34) | *N* = 181  -4.8 | *N* = 142  -5.0 (2.32) | *N* = 36  -4.1 | *N* = 33  -4.1 (2.41) | *N* = 38  -4.9 | *N* = 29  -5.4 (1.97) |
| Patient's assessment of nocturnal back pain^d^ | *N* = 182  -4.8 | *N* = 151  -5.0 (2.34) | *N* = 181  -4.8 | *N* = 142  -5.1 (2.56) | *N* = 36  -4.6 | *N* = 33  -4.7 (2.25) | *N* = 38  -4.5 | *N* = 29  -5.1 (2.51) |
| BASFI | *N* = 183  -3.8 | *N* = 152  -4.0 (2.18) | *N* = 181  -3.7 | *N* = 142  -3.9 (2.42) | *N* = 36  -3.3 | *N* = 33  -3.5 (2.74) | *N* = 38  -3.7 | *N* = 29  -4.5 (2.01) |
| ASDAS | *N* = 183  -2.1 | *N* = 150  -2.2 (1.18) | *N* = 181  -2.1 | *N* = 140  -2.2 (1.08) | *N* = 36  -1.8 | *N* = 32  -1.9 (1.03) | *N* = 38  -2.0 | *N* = 29  -2.2 (0.81) |
| BASMI_lin_ | *N* = 179  -0.9 | *N* = 154  -0.9 (1.11) | *N* = 176  -1.0 | *N* = 147  -1.1 (1.09) | *N* = 36  -0.5 | *N* = 31  -0.5 (0.87) | *N* = 38  -1.0 | *N* = 30  -1.1 (0.98) |
| MASES^e^ | *N* = 140  -3.4 | *N* = 122  -3.3 (3.31) | *N* = 132  -3.2 | *N* = 110  -3.6 (3.25) | *N* = 31  -2.8 | *N* = 27  -2.4 (3.63) | *N* = 21  -3.7 | *N* = 19  -3.9 (1.87) |
| ASQoL | *N* = 182  -7.3 | *N* = 153  -7.6 (5.06) | *N* = 180  -7.1 | *N* = 146  -7.5 (5.27) | *N* = 36  -5.3 | *N* = 33  -5.4 (5.70) | *N* = 38  -6.9 | *N* = 29  -7.2 (4.58) |
| ASAS HI | *N* = 182  -4.7 | *N* = 153  -4.9 (4.02) | *N* = 181  -4.9 | *N* = 146  -5.1 (4.07) | *N* = 36  -3.7 | *N* = 33  -3.7 (4.62) | *N* = 38  -5.3 | *N* = 29  -5.6 (3.77) |
| SPARCC score – SIJ | *N* = 142  -4.1 | *N* = 138  -4.4 (10.01) | *N* = 141  -3.3 | *N* = 131  -3.5 (8.92) | *N* = 29  -2.4 | *N* = 28  -2.0 (9.29) | *N* = 29  -0.8 | *N* = 25  -0.5 (5.52) |
| SPARCC score – Spine | *N* = 141  -4.6 | *N* = 137  -4.2 (10.27) | *N* = 141  -3.7 | *N* = 134  -4.1 (11.33) | *N* = 28  -3.6 | *N* = 27  -3.3 (9.01) | *N* = 29  -3.9 | *N* = 26  -4.6 (10.10) |

A total of 19 patients had both prior TNFi and IL-17i exposure.

SPARCC scores were based on magnetic resonance imaging from baseline, week 14, and week 104 (2-year reading), and from premature discontinuation visits or unscheduled visits that occurred after week 76 and prior to week 104.

^a^*N* = 150. ^b^*N* = 140. ^c^*N* = 32. ^d^NRS score 0–10. ^e^For patients with enthesitis (MASES > 0) at baseline.

*AO* as observed, *ASAS* Assessment of SpondyloArthritis international Society, *ASAS40* ≥ 40% improvement in three out of the four ASAS domains without worsening in the remaining domain, *ASAS20* ≥ 20% improvement in three out of the four ASAS domains without worsening in the remaining domain, *ASAS HI* ASAS Health Index, *ASAS PR* ASAS partial remission, *ASDAS* Axial Spondyloarthritis Disease Activity Score, *ASQoL* Ankylosing Spondylitis Quality of Life, *BASDAI50* ≥ 50% improvement in Bath Ankylosing Spondylitis Disease Activity Index, *BASFI* Bath Ankylosing Spondylitis Functional Index, *BASMI_lin_* Bath Ankylosing Spondylitis Metrology Index linear version, *bDMARD* biologic disease-modifying antirheumatic drug, *ID* inactive disease, *IL-17i* interleukin-17 inhibitor, *IR* inadequate response, *LDA* low disease activity, *MASES* Maastricht Ankylosing Spondylitis Enthesitis Score, *MMRM* mixed-effects model for repeated measures, *NRI* non-responder imputation, *NRS* numeric rating scale, *QD* once daily, *SD* standard deviation, *SIJ* sacroiliac joint, *SPARCC* SpondyloArthritis Research Consortium of Canada, *TNFi* tumor necrosis factor inhibitor

**Table S3** Subgroup analysis of selected efficacy endpoints at week 104 by type of prior bDMARD failure

| Subgroup | bDMARD lack of efficacy | | | | bDMARD intolerance | | | |
| --- | --- | --- | --- | --- | --- | --- | --- | --- |
| Treatment arm | **Placebo to upadacitinib  15 mg QD** | | **Continuous upadacitinib  15 mg QD** | | **Placebo to upadacitinib  15 mg QD** | | **Continuous upadacitinib  15 mg QD** | |
| Binary endpoints,  n (%) | **AO-NRI**  ***N* = 159** | **AO**  ***N* = 130** | **AO-NRI**  ***N* = 166** | **AO**  ***N* = 130** | **AO-NRI**  ***N* = 66** | **AO**  ***N* = 57** | **AO-NRI**  ***N* = 61** | **AO**  ***N* = 50** |
| ASAS40 | 93 (58.5) | 93 (71.5) | 104 (62.7) | 104 (80.0) | 40 (60.6) | 40 (70.2) | 43 (70.5) | 43 (86.0) |
| ASAS20 | 119 (74.8) | 119 (91.5) | 118 (71.1) | 118 (90.8) | 49 (74.2) | 49 (86.0) | 46 (75.4) | 46 (92.0) |
| ASDAS LDA (< 2.1) | 87 (54.7) | 87 (68.0)^a^ | 91 (54.8) | 91 (71.1)^a^ | 32 (48.5) | 32 (57.1)^b^ | 35 (57.4) | 35 (70.0) |
| ASDAS ID (< 1.3) | 54 (34.0) | 54 (42.2)^a^ | 49 (29.5) | 49 (38.3)^a^ | 19 (28.8) | 19 (33.9)^b^ | 18 (29.5) | 18 (36.0) |
| ASAS PR | 58 (36.5) | 58 (44.6) | 55 (33.1) | 55 (42.3) | 22 (33.3) | 22 (38.6) | 23 (37.7) | 23 (46.0) |
| BASDAI50 | 88 (55.3) | 88 (67.7) | 101 (60.8) | 101 (77.7) | 35 (53.0) | 35 (61.4) | 42 (68.9) | 42 (84.0) |
| Continuous endpoints, mean change from baseline | **MMRM** | **AO (SD)** | **MMRM** | **AO (SD)** | **MMRM** | **AO (SD)** | **MMRM** | **AO (SD)** |
| Patient's assessment of total back pain^c^ | *N* = 159  -4.7 | *N* = 130  -4.8 (2.39) | *N* = 166  -4.8 | *N* = 130  -5.1 (2.35) | *N* = 66  -4.1 | *N* = 57  -4.4 (2.38) | *N* = 61  -4.8 | *N* = 50  -5.1 (1.93) |
| Patient's assessment of nocturnal back pain^c^ | *N* = 158  -4.8 | *N* = 129  -5.0 (2.31) | *N* = 166  -4.8 | *N* = 130  -5.0 (2.52) | *N* = 66  -4.3 | *N* = 57  -4.6 (2.44) | *N* = 61  -4.9 | *N* = 50  -5.2 (2.22) |
| BASFI | *N* = 159  -3.8 | *N* = 130  -4.0 (2.31) | *N* = 166  -3.8 | *N* = 130  -4.1 (2.40) | *N* = 66  -3.6 | *N* = 57  -3.7 (2.29) | *N* = 61  -3.8 | *N* = 50  -4.1 (2.11) |
| ASDAS | *N* = 159  -2.1 | *N* = 128  -2.1 (1.21) | *N* = 166  -2.1 | *N* = 128  -2.2 (1.01) | *N* = 66  -1.8 | *N* = 56  -1.9 (1.22) | *N* = 61  -2.1 | *N* = 50  -2.3 (1.05) |
| BASMI_lin_ | *N* = 155  -0.8 | *N* = 130  -0.8 (1.12) | *N* = 162  -1.0 | *N* = 134  -1.0 (1.09) | *N* = 65  -1.0 | *N* = 57  -1.0 (1.01) | *N* = 59  -1.0 | *N* = 51  -1.0 (0.91) |
| MASES^d^ | *N* = 124  -3.3 | *N* = 105  -3.3 (3.53) | *N* = 117  -3.2 | *N* = 98  -3.6 (3.21) | *N* = 50  -3.5 | *N* = 44  -3.2 (2.99) | *N* = 42  -3.6 | *N* = 37  -3.8 (3.18) |
| ASQoL | *N* = 158  -7.1 | *N* = 131  -7.3 (5.06) | *N* = 165  -7.3 | *N* = 133  -7.8 (5.32) | *N* = 66  -6.6 | *N* = 58  -6.9 (5.38) | *N* = 61  -6.5 | *N* = 51  -6.8 (4.59) |
| ASAS HI | *N* = 158  -4.6 | *N* = 131  -4.8 (4.18) | *N* = 166  -5.1 | *N* = 133  -5.4 (4.06) | *N* = 66  -3.9 | *N* = 58  -4.0 (3.94) | *N* = 61  -4.7 | *N* = 51  -5.0 (3.78) |
| SPARCC score – SIJ | *N* = 124  -3.8 | *N* = 119  -3.4 (9.96) | *N* = 131  -3.1 | *N* = 119  -3.1 (8.34) | *N* = 51  -4.5 | *N* = 50  -5.2 (9.50) | *N* = 47  -2.9 | *N* = 43  -2.9 (9.08) |
| SPARCC score – Spine | *N* = 122  -4.6 | *N* = 117  -4.4 (10.35) | *N* = 130  -4.3 | *N* = 122  -5.0 (11.72) | *N* = 51  -3.9 | *N* = 50  -2.9 (9.13) | *N* = 47  -3.1 | *N* = 44  -2.5 (9.40) |

A total of 33 patients had both intolerance and lack of efficacy to prior bDMARDs.

SPARCC scores were based on magnetic resonance imaging from baseline, week 14, and week 104 (2-year reading), and from premature discontinuation visits or unscheduled visits that occurred after week 76 and prior to week 104.

^a^*N* = 128. ^b^*N* = 56. ^c^NRS score 0–10. ^d^For patients with enthesitis (MASES > 0) at baseline.

*AO* as observed, *ASAS* Assessment of SpondyloArthritis international Society, *ASAS40* ≥ 40% improvement in three out of the four ASAS domains without worsening in the remaining domain, *ASAS20* ≥ 20% improvement in three out of the four ASAS domains without worsening in the remaining domain, *ASAS HI* ASAS Health Index, *ASAS PR* ASAS partial remission, *ASDAS* Axial Spondyloarthritis Disease Activity Score, *ASQoL* Ankylosing Spondylitis Quality of Life, *BASDAI50* ≥ 50% improvement in Bath Ankylosing Spondylitis Disease Activity Index, *BASFI* Bath Ankylosing Spondylitis Functional Index, *BASMI_lin_* Bath Ankylosing Spondylitis Metrology Index linear version, *bDMARD* biologic disease-modifying antirheumatic drug, *ID* inactive disease, *LDA* low disease activity, *MASES* Maastricht Ankylosing Spondylitis Enthesitis Score, *MMRM* mixed-effects model for repeated measures, *NRI* non-responder imputation, *NRS* numeric rating scale, *QD* once daily, *SD* standard deviation, *SIJ* sacroiliac joint, *SPARCC* SpondyloArthritis Research Consortium of Canada

**Table S4** Treatment-emergent SAEs considered to be life threatening and/or with a reasonable possibility of being associated with the study drug

| **Patient sex, age (years)** | **Days after treatment onset** | **Type of event** | **Life threatening** | **Premature D/C** | **Reasonable possibility of association with study drug^a^** |
| --- | --- | --- | --- | --- | --- |
| Male, 40 | 319 | COVID-19 infection | Yes | Yes | No |
| Male, 36 | 122 | Complicated acute phlegmonous appendicitis | Yes | No | No |
|  | 122 | Local purulent peritonitis | Yes | No |  |
| Male, 43 | 95 | COVID-19 pneumonia | Yes | No | No |
| Male, 67 | 392 | Acute blood loss anemia | Yes | Yes | No |
|  | 392 | Upper GI hemorrhage | Yes | Yes | No |
|  | 392 | Esophageal varices | Yes | Yes | No |
| Male, 41 | 249 | Polytrauma | Yes (death) | N/A | No |
| Female, 30 | 101 | Pulmonary embolism | Yes | Yes | Yes |
| Male, 38 | 734 | Acute ST elevation MI | N/A^b^ | N/A^b^ | Yes |
| Female, 47 | 145 | Worsening of uterine fibroids | No | No | Yes |
| Male, 40 | 211 | Acute uveitis | No | No | Yes |
| Male, 62 | 463 | Disseminated herpes zoster | No | No | Yes |
| Female, 64 | 167 | Iatrogenic immunodeficiency‑associated lymphoproliferative disorder | No | No | Yes |

^a^Based on the investigator’s opinion of event causality. ^b^This event occurred 2 days after study drug completion and was determined by the investigator as non-life threatening; as it occurred within 30 days of the last dose, it was recorded as treatment emergent.

*D/C* discontinuation, *GI* gastrointestinal, *MI* myocardial infarction, *N/A* not applicable, *SAE* serious adverse event

**Table S5** Treatment-emergent malignancy and adjudicated cardiovascular events

| **Patient sex, age (years)** | **Days after treatment onset** | **Type of event** | **Premature D/C** | **Reasonable possibility of association with study drug^a^** |
| --- | --- | --- | --- | --- |
| **Malignancy** |  |  |  |  |
| Male, 60 | 14 | SCC of left tonsil | Yes | No |
| Male, 62 | 206 | BCC in the right nasal region | Yes | No |
| Male, 56 | 493 | Liver metastasis | Yes | No |
|  | 600 | Stage IV colon adenocarcinoma |  | No |
| Female, 54 | 483 | Relapse of superficial BCC of the chest | No | No |
| Female, 69 | 675 | BCC of the skin | No | No |
|  | 675 | SCC of the skin | No | No |
| **MACE** |  |  |  |  |
| Male, 38 | 734 | Acute ST elevation MI | No | Yes |
| Male, 47 | 241 | Cerebral hemorrhage at left basal ganglia | Yes | No |
| **VTE** |  |  |  |  |
| Male, 59 | 526 | DVT of lower left limb | Yes | Yes |
| Female, 30 | 101 | Pulmonary embolism | Yes | Yes |

^a^Based on the investigator’s opinion of event causality.

*BCC* basal cell carcinoma, *D/C* discontinuation, *DVT* deep vein thrombosis, *MACE* major adverse cardiovascular event, *MI* myocardial infarction, *SCC* squamous cell carcinoma, *VTE* venous thromboembolic event

| **Parameter, *n* (%)** | **Any upadacitinib 15 mg QD (*N* = 413)^b^** |
| --- | --- |
| Hemoglobin (g/L) |  |
| Grade 3 (< 80) | 1 (0.2) |
| Lymphocytes (10^9^/L) |  |
| Grade 3 (< 0.5–0.2) | 5 (1.2) |
| Grade 4 (< 0.2) | 0 |
| Neutrophils (10^9^/L) |  |
| Grade 3 (0.5–< 1.0) | 11 (2.7) |
| Grade 4 (< 0.5) | 0 |
| ALT (U/L) |  |
| Grade 3 (> 5.0–20.0 × ULN) | 6 (1.5) |
| Grade 4 (> 20.0 × ULN) | 0 |
| AST (U/L) |  |
| Grade 3 (> 5.0–20.0 × ULN) | 6 (1.5) |
| Grade 4 (> 20.0 × ULN) | 0 |
| Creatinine (μmol/L) |  |
| Grade 3 (> 3.0–6.0 × ULN or > 3.0 × BL) | 1 (0.2) |
| Grade 4 (> 6.0 × ULN) | 0 |

**Table S6** Patients with grade 3/4^a^ laboratory parameters at week 104

Laboratory abnormalities leading to treatment discontinuation included one event of anemia in one patient (not associated with the study drug according to the investigator), and one event of ALT elevation and AST elevation in the same patient (reasonable possibility of being associated with the study drug according to the investigator). Drug discontinuations were performed at the discretion of the investigator and were not dictated by specific laboratory cut-off values.

^a^Based on Common Terminology Criteria for Adverse Events, version 4.0. ^b^Number of patients with both baseline and post-baseline values for the specific criteria.

*ALT* alanine transaminase, *AST* aspartate transaminase, *BL* baseline, *QD* once daily; *ULN* upper limit of normal

**
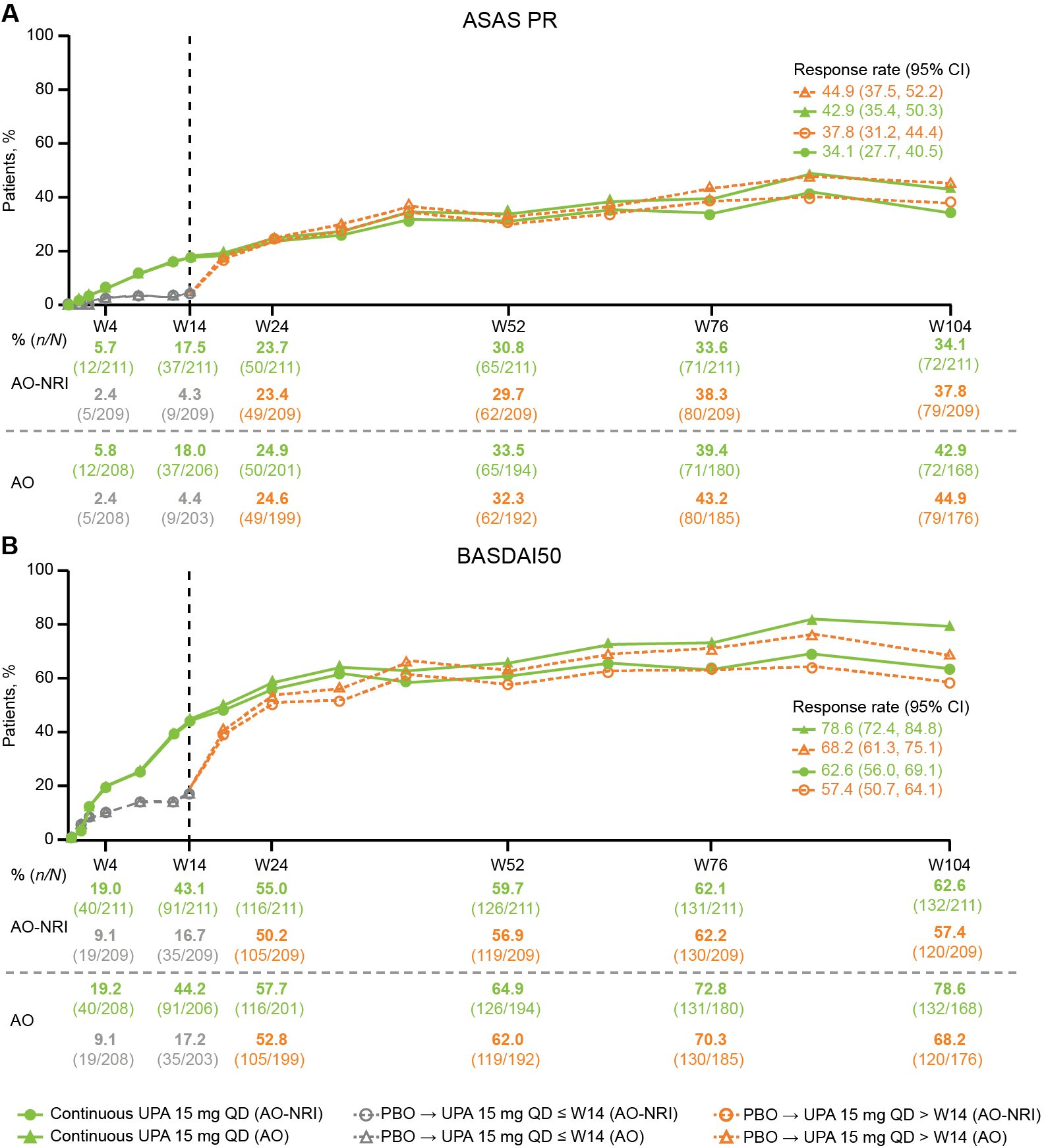
Fig. S1** Proportions of patients achieving ASAS PR (**A**) and BASDAI50 (**B**) responses through week 104 (AO-NRI and AO)

Patients who were initially randomized to placebo were switched to open-label upadacitinib at week 14.

*AO* as observed, *ASAS PR* Assessment of SpondyloArthritis international Society partial remission, *BASDAI50* ≥ 50% improvement in Bath Ankylosing Spondylitis Disease Activity Index, *CI* confidence interval, *NRI* non-responder imputation, *PBO* placebo, *QD* once daily, *UPA* upadacitinib, *W* week

**
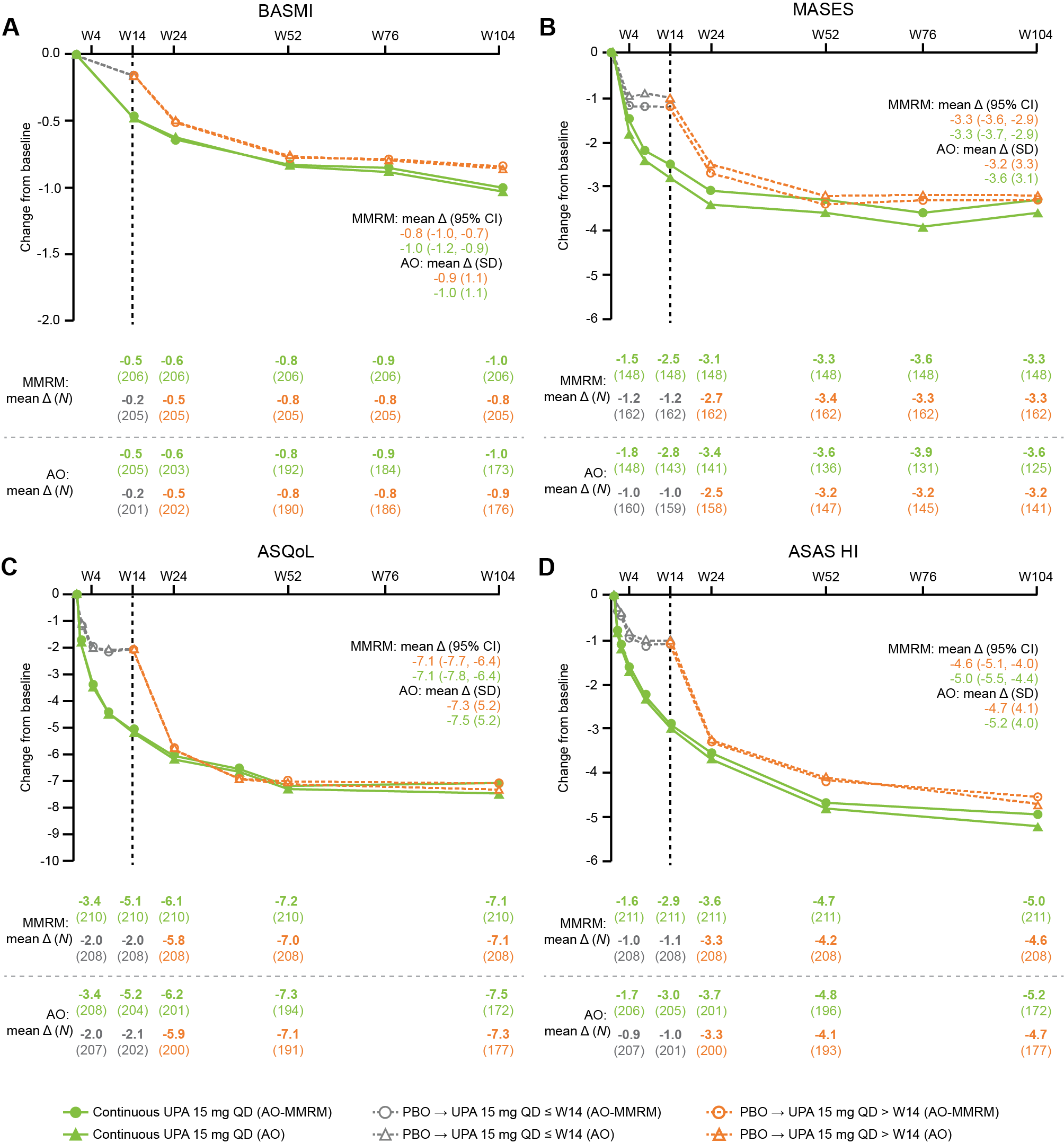
Fig. S2** Mean change from baseline in BASMI (**A**), MASES (**B**), ASQoL (**C**), and ASAS HI (**D**) through week 104 (AO-MMRM and AO)

Patients who were initially randomized to placebo were switched to open-label upadacitinib at week 14.

*Δ* change, *AO* as observed, *ASAS HI* Assessment of SpondyloArthritis international Society Health Index, *ASQoL* Ankylosing Spondylitis Quality of Life, *BASMI* Bath Ankylosing Spondylitis Metrology Index, *CI* confidence interval, *MASES* Maastricht Ankylosing Spondylitis Enthesitis Score, *MMRM* mixed-effects model for repeated measures, *PBO* placebo, *QD* once daily, *SD* standard deviation, *UPA* upadacitinib*, W* week

**
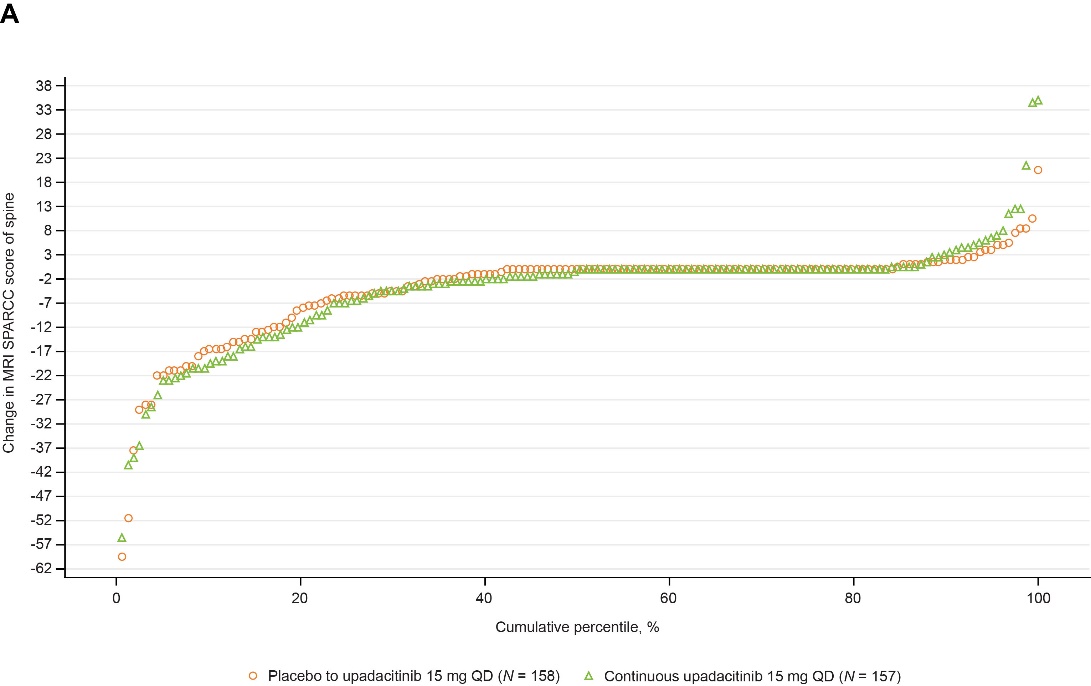
Fig. S3** Cumulative probability of change in MRI SPARCC score of spine (**A**), MRI SPARCC score of SIJs (**B**), and mSASSS (**C**) at week 104 by treatment sequence

**
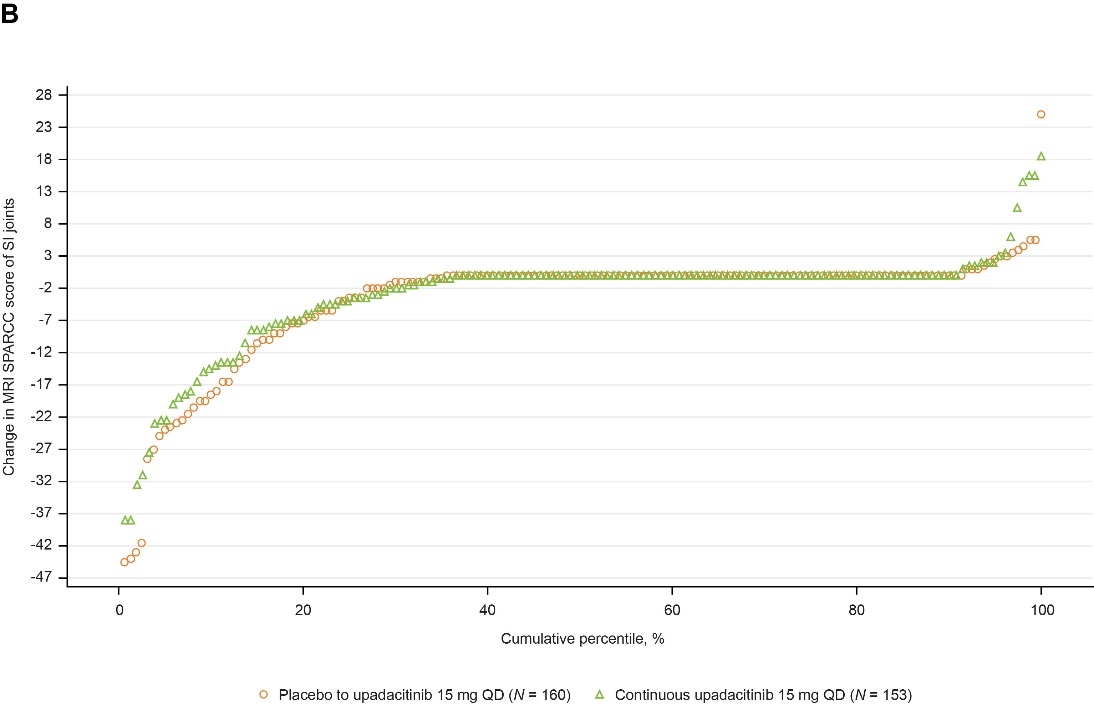
**


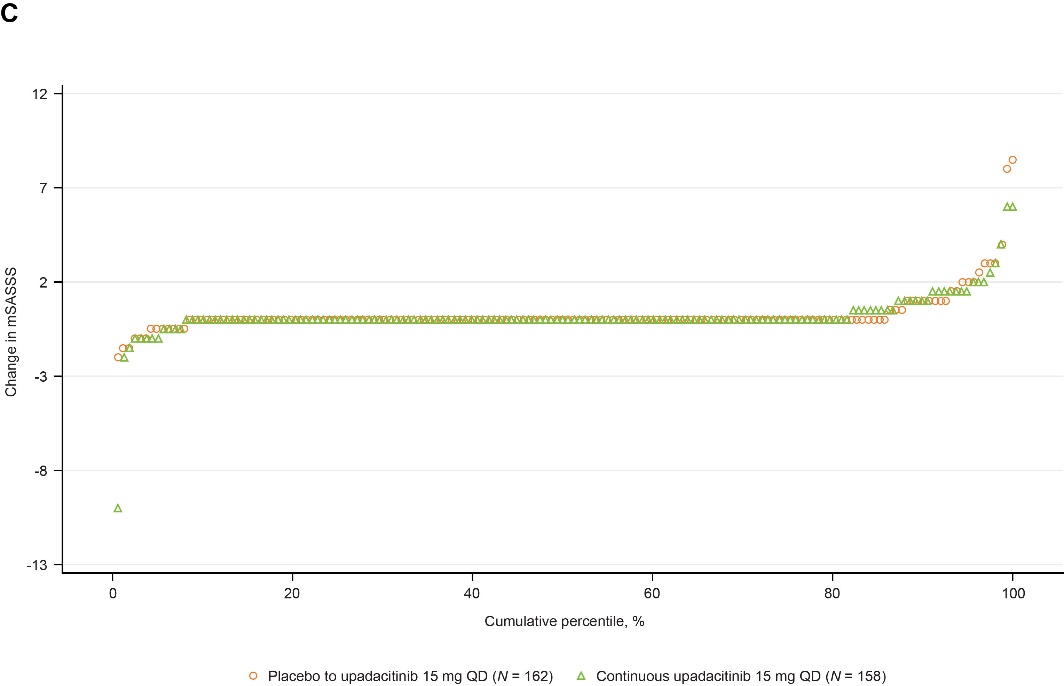
*MRI* magnetic resonance imaging, *mSASSS* modified Stoke Ankylosing Spondylitis Spinal Score, *QD* once daily, *SIJ* sacroiliac joint, *SPARCC* SpondyloArthritis Research Consortium of Canada

**
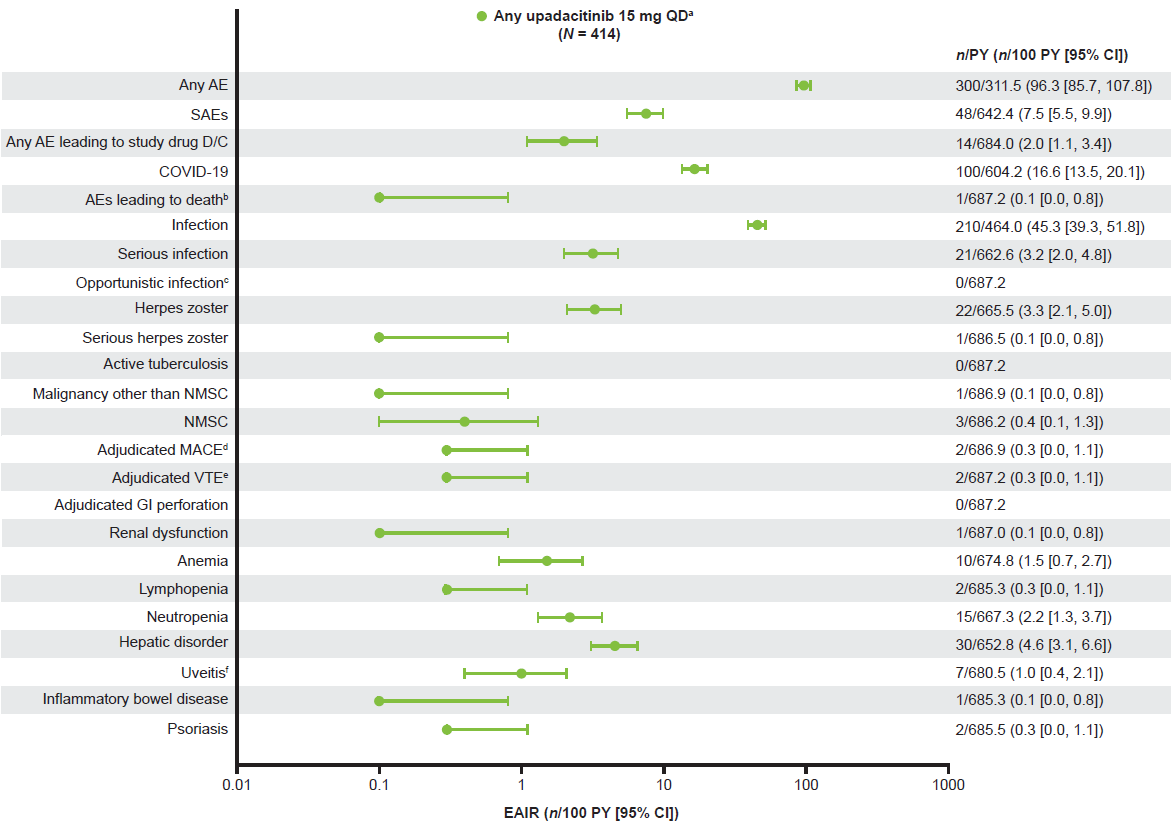
Fig. S4** EAIRs of TEAEs through week 104

^a^All patients who received ≥1 dose of upadacitinib 15 mg QD. ^b^One patient died due to polytrauma before week 52. ^c^Excluding tuberculosis and herpes zoster. ^d^Defined as cardiovascular death (includes acute myocardial infarction, sudden cardiac death, heart failure, cardiovascular procedure-related death, death due to cardiovascular hemorrhage, fatal stroke, pulmonary embolism, and other cardiovascular causes), non-fatal myocardial infarction, and non-fatal stroke. ^e^Includes deep vein thrombosis and pulmonary embolism (fatal and non-fatal). ^f^Includes uveitis, iritis, and iridocyclitis.

*AE* adverse event, *CI* confidence interval, *D/C* discontinuation, *EAIR* exposure-adjusted incidence rate, *GI* gastrointestinal, *MACE* major adverse cardiovascular events, *NMSC* non-melanoma skin cancer, *PY* patient-years, *QD* once daily, *SAE* serious AE, TEAE treatment-emergent AE, *VTE* venous thromboembolic events
